# Comparison of Anticancer Medication Use and Spending Under US Oncology Parity Laws With and Without Out-of-Pocket Spending Caps

**DOI:** 10.1001/jamahealthforum.2021.0673

**Published:** 2021-05-28

**Authors:** Stacie B. Dusetzina, Haiden A. Huskamp, Shelley A. Jazowski, Aaron N. Winn, Ethan Basch, Nancy L. Keating

**Affiliations:** 1 Department of Health Policy, Vanderbilt University School of Medicine, Nashville, Tennessee; 2 Vanderbilt-Ingram Cancer Center, Nashville, Tennessee; 3 Department of Health Care Policy, Harvard Medical School, Boston, Massachusetts; 4 Department of Health Policy and Management, Gillings School of Global Public Health, University of North Carolina at Chapel Hill; 5 Department of Population Health Sciences, Duke University School of Medicine, Durham, North Carolina; 6 School of Pharmacy, Medical College of Wisconsin, Milwaukee; 7 Center for Advancing Population Science, Medical College of Wisconsin, Milwaukee; 8 Cancer Center, Medical College of Wisconsin, Milwaukee; 9 Division of Oncology, Department of Medicine, University of North Carolina School of Medicine, Chapel Hill; 10 Associate Editor, *JAMA*; 11 Division of General Internal Medicine and Primary Care, Brigham and Women’s Hospital, Boston, Massachusetts

## Abstract

**Importance:**

By 2020, nearly all states had adopted oncology parity laws in the US, ensuring that patients in fully insured private health plans pay no more for orally administered anticancer medications (OAMs) than infused therapies. Between 2013 and mid-2017, 11 states implemented parity with out-of-pocket spending caps, which may further reduce patient out-of-pocket spending.

**Objective:**

To compare OAM uptake and out-of-pocket and health plan spending on OAMs in states with parity with and without spending caps, as well as to assess out-of-pocket spending for caps that apply predeductible vs postdeductible.

**Design, Setting, and Participants:**

This cohort study analyzed OAM users enrolled in commercial health plans offered by Aetna, Humana, and United Healthcare in the US from 2011 to 2017, aggregated by the Health Care Cost Institute, using difference-in-difference-in-differences (DDD) analysis. Data analysis was conducted between June and August 2020.

**Exposures:**

Time (before vs after parity), whether the state parity law included an out-of-pocket spending cap, and whether the plan was fully insured (subject to parity) or self-funded (not subject to parity). Among states with caps, out-of-pocket spending was also compared by whether the cap was applied predeductible and postdeductible vs only postdeductible.

**Main Outcomes and Measures:**

Monthly OAM prescription fills per 100 000 enrollees, per-OAM prescription-fill out-of-pocket spending, and annual per-user health plan spending on OAMs.

**Results:**

In this study of 23 states (11 with caps and 12 without) and 207 579 OAM prescription fills, caps were associated with a modest increase in OAM use (DDD, 7.40 [95% CI, 3.41-11.39] per 100 000 enrollees). There was no difference in mean out-of-pocket spending comparing fully insured and self-funded enrollees in states with vs without caps (DDD, −$17 [95% CI, −$57 to $24), but caps were associated with lower spending among OAM users in the 95th percentile of out-of-pocket spending by $831 (95% CI, −$871 to −$791) per OAM prescription fill. Caps applied predeductible were associated with greater out-of-pocket savings relative to caps applied only postdeductible. This included per-OAM prescription-fill savings at the 75th, 90th, and 95th percentiles. Postparity, mean annual spending on OAMs among users was $113 589 in states without caps and $102 252 in states with caps, with no differences between groups (DDD, $9799 [95% CI, −$4230 to $23 829).

**Conclusions and Relevance:**

In this cohort study, among states adopting oncology parity laws between 2013 and 2017, mean out-of-pocket spending per OAM prescription fill and mean health plan spending among OAM users was similar in states with and without caps. However, enrollees in states with parity plus out-of-pocket caps had greater reductions in out-of-pocket spending among the highest spenders. Caps may offer improved financial protection for the highest spenders without increasing mean health plan spending on OAMs.

## Key Points

**Question:**

How does orally administered anticancer medication (OAM) use and spending differ among states that adopted parity with vs without out-of-pocket spending caps?

**Findings:**

In this cohort study of 23 states and 207 579 OAM prescription fills, out-of-pocket spending caps were associated with higher OAM use and lower out-of-pocket spending by $831 per OAM prescription fill among the highest spenders. Savings were larger for enrollees in states with caps that applied predeductible and postdeductible vs those that applied postdeductible only.

**Meaning:**

Caps may offer improved financial protection for the highest spenders without increasing mean health plan spending on OAMs.

## Teaser

This cohort study compares orally administered anticancer medication uptake, out-of-pocket spending, and health plan spending in states with parity with and without spending caps.

## Introduction

The number of orally administered anticancer medications (OAMs) has increased rapidly in recent years, from 13 in 2010 to more than 50 by 2019.^1^ High prices for OAMs—combined with greater use of deductibles and coinsurance in commercial health plans^2^—have raised concerns regarding access to medications even among insured populations. Specifically, higher out-of-pocket costs can result in patients abandoning medications^3,4,5^ or taking less than prescribed,^6^ even for highly effective anticancer drugs.

Because OAMs are generally covered under a plan’s pharmacy benefit, advocates have voiced concern that patients may face higher out-of-pocket costs for these drugs than they would for drugs offered under a plan’s medical benefit (ie, infused chemotherapies).^7^ These concerns have resulted in widespread adoption of oral anticancer drug parity laws since 2008. By early 2019, all but 7 states had adopted oral oncology parity laws, and federal legislation to extend parity^8^ has been introduced in both the US House and Senate.

Prior to 2013, parity laws typically required plans to offer orally administered and infused anticancer treatments under the same cost-sharing arrangements, without dictating a limit on out-of-pocket expenditures. These early laws led to modest savings for many OAM users but not for the highest spenders,^9^ who saw increases in OAM spending. Since 2013, 11 states adopting parity have also included out-of-pocket spending caps, ranging from $50 to $300 for approximately 1 month of OAM supply. In addition, 6 of these states required that caps apply both before and after a deductible is met (Table 1). Whether these laws are associated with greater medication uptake and lower out-of-pocket spending relative to traditional parity laws is unknown.

**Table 1.** States With Oncology Parity Laws With and Without Out-of-Pocket Caps Enacted 2013-2017

State name	Prescription fill, No.		Cap amount, $	Cap applies predeductible^a^	Parity date
	Fully insured	Self-funded			
**States with caps**					
Total	107 632	56 306	NA	NA	NA
California	16 757	13 015	200	No	1/1/15
Florida	31 922	12 105	50	Yes	7/1/14^b^
Georgia	16 976	8042	200	Yes	1/1/15
Kentucky	6440	2884	100	No	1/1/15
Louisiana	3820	2101	100	No	1/1/13
Missouri	9057	2961	75	Yes	1/1/15
Nevada	1869	731	100	Yes	1/1/15
Ohio	9808	8394	100	No	1/1/15
Oklahoma	2132	824	100	Yes	11/1/13
Utah	977	860	300	Yes	10/1/13
Wisconsin	7661	4036	100	No	1/1/15
**States without caps**					
Total	26 822	16 819	NA	NA	NA
Alaska	114	929	NA	NA	1/1/17
Arizona	9917	4237	NA	NA	1/1/16
Delaware	262	156	NA	NA	1/1/13
Maine	775	1148	NA	NA	1/1/15
Massachusetts	677	1701	NA	NA	5/1/13
Mississippi	3406	862	NA	NA	1/1/15
North Dakota	160	130	NA	NA	8/1/15
Pennsylvania	8603	6292	NA	NA	1/1/16
Rhode Island	834	828	NA	NA	1/1/14
South Dakota	120	110	NA	NA	1/1/16
West Virginia	1748	320	NA	NA	1/1/16
Wyoming	206	106	NA	NA	7/1/15

Abbreviation: NA, not applicable.

^a^ The deductible is included in the cap for high-deductible health plans and does not need to be paid before the cap applies. Differences in sample size per state are due to variations in both state population and insurance coverage by a Health Care Cost Institute–contributing plan (Aetna, Humana, United Healthcare) over the study period.

^b^ In Florida, parity went into effect on July 1, 2014, but it affected plans renewing on or after this date; because most plans renew on a calendar year, January 1, 2015, was maintained as the effective date for Florida, and sensitivity analyses were conducted excluding Florida to ensure that the results were robust to this modification.

In this cohort study, we compared OAM uptake and out-of-pocket spending for patients in fully insured plans (subject to parity) with those in self-funded plans (not subject to parity) by the presence or absence of an out-of-pocket spending cap. We hypothesized that fully insured enrollees in states that include out-of-pocket spending caps in addition to parity would have greater use and lower out-of-pocket spending on OAMs than those in states with parity alone. We also assessed differences in out-of-pocket spending among states with spending caps applied both predeductible and postdeductible vs only postdeductible. Finally, we assessed the association of caps with mean annual per-user OAM spending by health plans to evaluate whether caps increase health plan spending on these drugs.

## Methods

### Data and Patients

We used 2011-2017 national health plan claims for privately insured members of Aetna, Humana, and UnitedHealthcare, aggregated by the Health Care Cost Institute. We included OAM prescription fills (eTable 1 in the Supplement) from states that implemented parity between January 1, 2013, and June 30, 2017. We restricted prescription fills to this time frame because none of the parity laws implemented before January 1, 2013, included out-of-pocket spending caps; including earlier years of data would likely confound estimates of differences between parity laws with and without caps because the number of treatment options, drug prices, and coverage policies have changed over time. We included OAM prescription fills for enrollees younger than 65 years (because parity laws do not extend to Medicare), restricting to the 24 months before and after parity in each state. The unit of analysis is the person-fill with each prescription fill representing approximately 1 month of OAM supply. This study was approved by the Vanderbilt University Institutional Review Board, and need for patient consent was waived owing to use of deidentified data. The authors followed the Strengthening the Reporting of Observational Studies in Epidemiology (STROBE) reporting guidelines for cohort studies. Data analysis was conducted between June and August 2020.

### Key Outcomes

We measured monthly OAM prescription fills per 100 000 enrollees and out-of-pocket spending per OAM prescription fill (including co-payments, coinsurance, and deductibles). We examined changes in mean out-of-pocket spending and the distribution of out-of-pocket spending among OAM prescription fills. We also measured mean annual OAM spending by health plans among OAM users.

### Key Independent Variables

We included indicators for time (before vs after parity), whether the state parity law included an out-of-pocket spending cap, and whether the plan was fully insured (subject to parity) or self-funded (not subject to parity). Main effect sizes and 2-way and 3-way interactions were modeled. The 3-way interaction (the difference-in-difference-in-differences [DDD] estimator) indicates whether differences in utilization or spending postparity are similar among fully insured plans in states with and without caps, adjusting for trends among self-funded plans over the same period.

We also examined whether out-of-pocket spending varied by the cap applied predeductible and postdeductible vs only postdeductible. For these models, we compared changes by group preparity and postparity among fully insured enrollees and self-funded enrollees in states with parity and caps (excluding states with parity alone from these analyses).

### Statistical Analysis

We used a DDD design^10,11^ for estimating OAM prescription fills per 100 000 enrollees via a generalized estimating equation with an identity link and normal distribution. We tested for differential trends in monthly OAM prescription use during the 24 months preparity and did not find evidence for differential trends.

Next, we estimated changes in mean out-of-pocket spending and annual per-user OAM spending by health plans using generalized estimating equations with an identity link and gamma distribution, controlling for repeated OAM prescription fills among enrollees. Annual spending included any OAM spending during the year an OAM was filled, regardless of prior or subsequent enrollment. This allows enrollees to enter and exit the cohort (including death), providing an average of health plan spending on OAMs across all OAM users each year. We also used quantile regression^12,13^ to examine changes in the distribution of out-of-pocket spending. In addition to accounting for time-invariant characteristics through our modeling approach, we estimated propensity score–weighted (adjusted) models, including age group, sex, and quarter when the prescription was filled. We used a multinomial logistic regression model to generate the propensity score for each group (eMethods in the Supplement). We excluded year and drug filled from the propensity score model because these variables are closely tied to group membership and thus not recommended for inclusion in difference-in-differences (DD) models for assessing policy change.^14^

We estimated 2 sets of models for each out-of-pocket spending measure: (1) DD models stratified by the presence or absence of an out-of-pocket spending cap (estimating changes preparity and postparity in fully insured members vs self-funded members in the same state), and (2) DDD models that compared changes in states with vs without caps. The former allows us to determine how spending has changed over time within states with parity alone and, separately, for states implementing parity with caps. The latter allows us to compare across parity implementation strategies (caps vs no caps). We estimated separate propensity score models for each contrast and produced unadjusted and propensity score–weighted estimates and 95% CIs, with 2-sided *P* < .05 considered statistically significant. All spending was inflation adjusted to 2017 dollars using the medical component of the consumer price index. Unadjusted results were similar to the propensity score–weighted estimates. Finally, among states with caps, we replicated our out-of-pocket spending analyses to test whether out-of-pocket spending differed among states with caps that applied predeductible and postdeductible vs postdeductible only. All analyses were conducted using SAS, version 9.4 (SAS Institute).

### Sensitivity Analyses

We tested alternate model specifications for the link functions and distributions for continuous outcomes; these changes did not meaningfully alter our estimates. Second, while most states that implemented parity during the study period did so between 2013 and 2015, 5 states implemented parity in later years (2016 or 2017) and had fewer than 2 years of follow-up. We restricted analysis to states passing parity between 2013 and 2015 and found no differences in the results. Third, to ensure findings were not driven by a few large states, we excluded California, Florida, and Georgia because they contributed more than half of the observations within fully insured plans with caps. Excluding these states did not change the results. Fourth, 2 generic OAMs were approved during the study period (imatinib, the generic for Gleevec, in 2015 and capecitabine, the generic for Xeloda, in 2013), which may have lowered out-of-pocket costs for these treatments independent of parity laws owing to lower prices through generic competition or health plan policies that use lower co-payments for generic drugs. Sensitivity analyses excluding the branded and generic versions of these products were nearly identical to the primary analysis. Finally, in the primary model of plan spending we focused on OAM spending alone. In sensitivity analyses, we included total outpatient prescription drug spending (OAMs and other prescriptions) with similar results (eFigure 2 in the Supplement).

## Results

Of the 23 states and 207 579 person-fills for OAMs, 79% were from states with parity and out-of-pocket spending caps (n = 11), and 21% were from states with parity alone (n = 12). Approximately 65% of OAM prescription fills were among fully insured plan members subject to parity, and 35% were among self-funded plan members exempt from parity (within-state controls) (Table 1). Among states with caps, 6 required that the cap apply predeductible and postdeductible, and 5 required that the cap apply postdeductible only.

### OAM Use

We observed increased OAM use over time among all fully insured enrollees, with greater increases among those in states with parity and caps (from 33.2 to 50.1 OAM prescription fills per 100 000 enrollees) vs those with parity alone (from 33.2 to 47.5 OAM prescription fills per 100 000 enrollees) (Table 2 and eFigure 1 in the Supplement). Controlling for trends in self-funded plans over the same period, we observed an increase of 7.4 OAM prescription fills per month per 100 000 enrollees postparity among those in parity states with caps relative to those in parity states without caps (DDD, 7.40; 95% CI, 3.41-11.39; relative increase of 22%).

**Table 2.** Changes in OAM Use per 100 000 Enrollees Preparity and Postparity by Funding Status and Out-of-Pocket Spending Cap Use^a^

OAM use	Fully insured		Self-funded		DD estimate: fully insured vs self-funded		DDD estimate: caps vs no cap fully insured vs self-funded	
	Preparity	Postparity	Preparity	Postparity	DD (95% CI)	*P* value	DDD (95% CI)	*P* value
OAM fills per month with caps	33.2	50.1	22.1	26.2	17.0 (13.7-20.3)	<.001	7.4 (3.4-11.4)	<.001
OAM fills per month without caps	33.2	47.5	22.1	28.4	9.6 (7.3-11.9)	<.001	NA	NA

Abbreviations: DD, difference in differences; DDD, difference in difference in differences; NA, not applicable; OAM, orally administered anticancer medication.

^a^ Analysis of 2011-2017 Health Care Cost Institute claims data. There was not evidence for differential baseline trends preparity, which supports the parallel trends assumption (trend by cap and plan type, 0.10; 95% CI, −0.01 to 0.21). Models were adjusted using inverse probability of treatment propensity score weights, controlling for age, sex, and the quarter in which the prescription was filled.

### Out-of-Pocket Spending

#### States With Caps vs States Without Caps

Mean per-OAM prescription fill out-of-pocket spending decreased by $87 (DD, −$87; 95% CI, −$115 to −$60) among states with parity and caps, and by $69 (DD, −$69; 95% CI, −$99 to −$39) among states with parity without caps for fully insured enrollees as compared with self-funded enrollees in those states over the same period (Table 3). There was no difference in mean out-of-pocket spending comparing states with vs without caps (DDD, −$17; 95% CI, −$57 to $24).

**Table 3.** Changes in the Distribution of Out-of-Pocket Spending per OAM Fill Preparity and Postparity by Plan Funding, Stratified by Presence vs Absence of an Out-of-Pocket Spending Cap

Measure	Fully insured		Self-funded		Adjusted DD estimate: fully insured vs self-funded (95% CI)	Adjusted DDD estimate: cap vs no cap fully insured vs self-funded (95% CI)
	Preparity	Postparity	Preparity	Postparity		
**Parity with cap, $**						
OAM fill total, No.	40 581	65 997	28 268	27 899	NA	NA
Mean^a^	276	202	138	151	−87 (−115 to −60)	−17 (−57 to 24)
25th Percentile	0	0	0	0	0	0
50th Percentile	39	0	37	26	−28 (−28 to−28)	9 (9 to 9)
75th Percentile	167	77	67	61	−85 (−86 to −83)	12 (−14 to −11)
90th Percentile	559	530	121	133	−42 (−51 to −33)	1 (−10 to 13)
95th Percentile	1635	720	254	318	−981 (−1016 to −947)	−831 (−871 to −791)
**Parity without cap, $**						
OAM fill total, No.	10 639	16 143	7503	9316	NA	NA
Mean^a^	233	186	89	112	−69 (−99 to −39)	NA
25th Percentile	0	0	0	0	0	NA
50th Percentile	38	0	28	26	−36 (−36 to −36)	NA
75th Percentile	113	42	50	51	−71 (−74 to −67)	NA
90th Percentile	535	510	106	122	−35 (−44 to −27)	NA
95th Percentile	862	746	218	250	−161 (−227 to −95)	NA

Abbreviations: DD, difference in differences; DDD, difference in difference in differences; OAM, orally administered anticancer medication; NA, not applicable.

^a^ Based on analysis of Health Care Cost Institute claims from 2011 to 2017, means were estimated using a generalized estimating equation with an identity link and gamma distribution. Quantile regression was used to estimate changes in the distribution of spending at the 25th, 50th, 75th, 90th, and 95th percentiles. Models were adjusted using inverse probability of treatment propensity score weights, controlling for age, sex, and the quarter in which the prescription was filled.

We observed statistically significant savings for fully insured plan members after parity for both states with caps and states without caps as compared with self-funded members within the state (Table 3). At the 90th percentile of out-of-pocket spending or less, we found no difference in out-of-pocket spending comparing states with vs without caps. However, fully insured enrollees with the highest levels of out-of-pocket spending (95th percentile) saved $831 more per OAM prescription fill in states with parity plus out-of-pocket spending caps than those in states with traditional parity laws without caps (DDD, −$831; 95% CI, −$871 to −$791), controlling for trends in self-funded plans over the same period.

#### States With Caps Applied Predeductible vs Only Postdeductible

**Figure 1.**
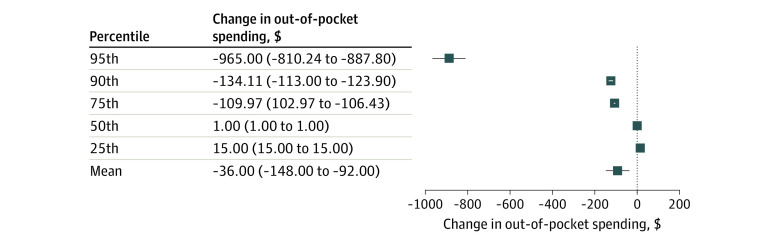
Changes in Out-of-Pocket Spending for OAM Fills Among Fully Insured Plan Members in States With Caps Predeductible and Postdeductible vs Only Postdeductible, Controlling for Trends in Self-Funded Plans In this analysis of 2011-2017 Health Care Cost Institute claims data, means were estimated using a generalized estimating equation with an identity link and gamma distribution. Models were adjusted using inverse probability of treatment propensity score weights, controlling for age, sex, and the quarter in which the prescription was filled. OAM indicates orally administered anticancer medication.

When comparing out-of-pocket spending trends among states with caps, we found that fully insured enrollees in states with caps that applied predeductible had greater declines in out-of-pocket spending than those with caps that applied only postdeductible. We observed mean savings of $92 (95% CI, −$148 to −$36; *P* < .001) per OAM prescription fill and savings of $106 (95% CI, −$103 to −$110), $124 (95% CI, −$114 to −$134), and $888 (95% CI, −$810 to −$965) at the 75th, 90th, and 95th percentiles, respectively (Figure 1 and eTable 2 in the Supplement).

### Mean Annual OAM Spending by Health Plans: States With Caps vs States Without Caps

**Figure 2.**
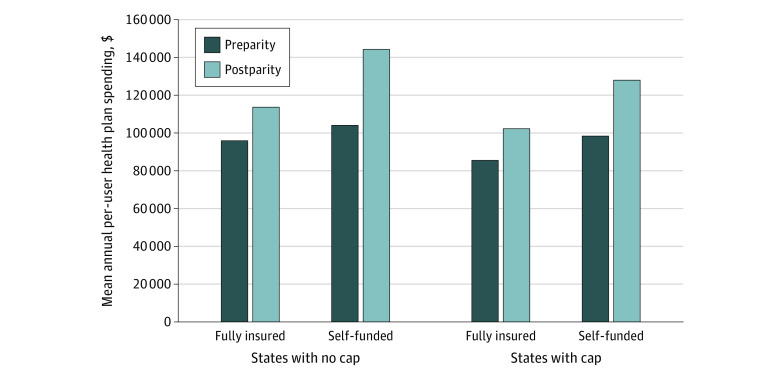
Mean Annual Per-User Health Plan Spending on OAMs Preparity and Postparity by Plan Funding and Presence vs Absence of an Out-of-Pocket Spending Cap In this analysis of 2011-2017 Health Care Cost Institute claims data, means were estimated using a generalized estimating equation with an identity link and normal distribution. Propensity score–weighted difference-in-difference-in-differences models (controlling for age, sex, and the quarter in which the prescription was filled) estimated a nonstatistically significant additional $9799 per person-year in annual total orally administered anticancer medication (OAM) spending for those in fully insured plans with caps relative to those in fully insured plans without caps, controlling for changes among self-funded members over the same period (difference in difference in differences, $9799; 95% CI, −$4230 to $23 829).

Mean annual outpatient OAM spending among those with any OAM prescription fills increased among fully insured plan members from $95 920 preparity to $113 589 postparity in states without caps and from $85 507 to $102 252 in states with caps. After controlling for changes among self-funded members, we estimated a nonstatistically significant increase of $9799 per person-year in annual total drug spending for those in fully insured plans with caps relative to those in fully insured plans without caps (DDD, $9799; 95% CI, −$4230 to $23 829; Figure 2).

## Discussion

Among enrollees in states adopting oncology parity laws between 2013 and 2017, mean out-of-pocket spending per OAM prescription fill declined similarly in states with and without caps. However, enrollees in states with parity plus out-of-pocket caps had greater reductions in out-of-pocket spending among the highest spenders (95th percentile). Despite modestly increased use of OAM prescriptions per 100 000 enrollees in states whose parity laws implemented caps, we found no evidence that parity with caps increased mean annual per-user health plan spending on OAMs relative to parity without caps. We also found that, among states with caps, those that required that caps apply predeductible resulted in savings for individuals at the mean and at the 75th, 90th, and 95th percentiles of out-of-pocket spending relative to states that required that caps apply only postdeductible.

Our prior work evaluating state parity laws and out-of-pocket spending between 2008 and 2012 found modest savings for most enrollees but increases in out-of-pocket spending for individuals with high spending.^9^ In fact, savings appeared to be achieved primarily through plans lowering co-payments from approximately $30 to $0 per OAM prescription fill. Our previous findings differ from the current study, where we observed that parity (with or without spending caps) was associated with reduced out-of-pocket spending, even for the highest spenders. This difference could be related to the more recent time period, for which more OAMs were available and prices were higher, differences in plan generosity for states that were early vs late adopters of parity, or changes in insurance design over time (eg, growing use of deductibles or Affordable Care Act requirements that plans include prescription drugs in out-of-pocket limits). Indeed, the level of preparity out-of-pocket spending has increased substantially among fully insured plan members over time. For states passing parity between 2008 and 2012, the 90th and 95th percentiles of preparity out-of-pocket spending among fully insured adults were $105 and $184 per OAM prescription fill, respectively,^9^ vs $535 and $862 among states without caps and $559 and $1635 among states with caps in the current study (for years 2013-2017).

While out-of-pocket savings were achieved for all enrollees subject to parity in the current study, the results suggest that caps combined with traditional parity could help to ensure that patients with the highest out-of-pocket spending are more directly targeted than under traditional parity laws. Additionally, states that required caps to apply both predeductible and postdeductible further shielded patients from very high out-of-pocket spending on OAMs relative to states that required that caps apply only postdeductible. Prior work has shown high rates of cost-related nonadherence of OAMs in the face of high out-of-pocket expenditures,^3,4,15,16^ as would be expected under a high-deductible health plan. Given the increased number of enrollees with deductibles, the growth in the amount required to meet the deductible, and the long-term use of many OAMs today, these additional measures may be increasingly important for ensuring adequate cost protections and medication adherence for patients.

It is important to recognize the growing costs of OAMs for health plans. While we did not observe a statistically significant increase in mean annual medication spending per OAM prescription user for those subject to parity and caps relative to those subject to parity alone, the magnitude of per-user spending on OAMs was high and growing among all enrollees. In the postparity period, mean annual OAM spending per anticancer medication user ranged from $106 073 to $152 065. While cancer—and OAM use—is relatively rare among commercially insured adults, these increases in prescription-drug spending are considerable over a relatively short time frame. Higher spending on OAMs over time may be due to use of OAMs instead of infused therapies, greater adherence to OAMs, or price increases. Efforts to improve patient access to anticancer medication should be paired with efforts aimed at managing total drug and health care spending. This is particularly important because even anticancer drugs with low or no clinical benefit typically have very high prices for payers and patients, which may increase spending without improving outcomes.^17^

### Limitations

Our study has limitations. First, we cannot determine if greater access to care or overuse was associated with the increased uptake of OAM prescriptions observed. We focused only on OAM users and did not evaluate use of infused therapies, other treatment modalities, or health outcomes among the cohort. Future studies with richer clinical data should evaluate whether high out-of-pocket spending for OAM prescriptions results in therapeutic substitution (from oral to infused therapies when both options are available and appropriate) or results in poor outcomes for patients. Second, we could not observe plan-level utilization management policies (prior authorization, step therapy, and quantity limits) and use of manufacturer coupons or other forms of patient co-payment assistance, nor could we account for patients who never filled a prescription owing to cost (their prescriptions are unobserved). Although coupons and co-payment assistance may lower actual out-of-pocket spending, the difference in out-of-pocket spending (our primary interest) would still be valid assuming similar program use among self-funded and fully insured patients in the same state and time period. Third, we studied patients in 3 national health plans; results may not be generalizable to other commercial insurers. Although the sample size was large, there were many more patients observed from states that implemented caps relative to those with traditional parity during the study period. This was partly because the traditional parity group was composed of less densely populated states; these states may have also had less payer representation. Fourth, most states requiring out-of-pocket spending caps required co-payments of $100 or $200 per OAM prescription fill, but caps varied from $50 in Florida to $300 in Utah, and these latter states both applied caps predeductible and postdeductible, complicating comparisons of how the cap level is associated with outcomes. Prior work suggests that paying more than $100 is an important indicator of prescription drug abandonment,^3^ though we were not able to test that association in this study. Finally, we focused only on individuals in states that implemented parity between 2013 and 2017, and did not include a nonparity control group because only 7 states have not yet adopted parity laws. Instead, self-funded plan members (exempt from parity) were used to determine changes expected in absence of parity.

## Conclusions

In this cohort study of oral oncology parity laws, out-of-pocket spending caps were associated with modestly increased OAM use, as well as similar reductions in mean out-of-pocket spending per OAM prescription fill and mean health plan spending on OAMs compared with traditional parity laws alone. However, out-of-pocket caps reduced out-of-pocket spending among those with the highest spending preparity, with the greatest savings observed among states that applied caps both predeductible and postdeductible (rather than postdeductible alone). US Congress has proposed federal reforms related to oncology parity in recent years, along with many other measures aiming to improve access to high-priced drugs. Federal parity legislation would represent an important advance from state efforts because it would extend parity to individuals in states that do not currently have parity and to self-funded plan members across all states. The present results suggest that federal parity efforts should include both traditional parity and an out-of-pocket spending cap— ideally applied predeductible and postdeductible—to ensure that those most in need of financial protections benefit from parity legislation.

## Notes

Supplement. **eMethods.** Description of Propensity Score Weighting Approach **eTable 1.** Anticancer Medication Fills Observed in the 24 Months Pre- and Post-Parity by Product Name **eFigure 1.** Changes in Orally-Administered Anticancer Medication Use Per 100,000 Enrollees Pre- and Post-Parity by Funding Status and Out-of-Pocket Spending Cap Use **eFigure 2.** Changes in Mean Annual Prescription Drug Spending by Health Plans Pre-and Post-Parity by Plan Funding, and Presence Versus Absence of an Out-of-Pocket Spending Cap **eTable 2.** Changes in the Distribution of Out-of-Pocket Spending Per Fill Pre-and Post-Parity by Plan Funding, Stratified by Whether Caps Applied Pre- and Post-Deductible or Post-Deductible Alone (Among States with Caps)
