# Supplementary material for: Comparison of Anticancer Medication Use and Spending Under US Oncology Parity Laws With and Without Out-of-Pocket Spending Caps
Source: JAMA Health Forum. 2021 May 28;2(5):e210673. doi: 10.1001/jamahealthforum.2021.0673 (PMC8796987; doi:10.1001/jamahealthforum.2021.0673)
Supplement: Supplement. — eMethods. Description of Propensity Score Weighting Approach eTable 1. Anticancer Medication Fills Observed in the 24 Months Pre- and Post-Parity by Product Name eFigure 1. Changes in Orally-Administered Anticancer Medication Use Per 100,000 Enrollees Pre- and Post-Parity by Funding Status and Out-of-Pocket Spending Cap Use eFigure 2. Changes in Mean Annual Prescription Drug Spending by Health Plans Pre-and Post-Parity by Plan Funding, and Presence Versus Absence of an Out-of-Pocket Spending Cap eTable 2. Changes in the Distribution of Out-of-Pocket Spending Per Fill Pre-and Post-Parity by Plan Funding, Stratified by Whether Caps Applied Pre- and Post-Deductible or Post-Deductible Alone (Among States with Caps) [file jamahealthforum-e210673-s001.pdf]

Supplemental Online Content

Dusetzina SB, Huskamp HA, Jazowski SA, Winn AN, Basch E, Keating NL. Comparison of anticancer medication use and spending under US oncology parity laws with and without out-of-pocket spending caps. *JAMA Health Forum*. 2021;2(5):e210673. doi:10.1001/jamahealthforum.2021.0673

eMethods. Description of Propensity Score Weighting Approach

eTable 1. Anticancer Medication Fills Observed in the 24 Months Pre- and Post-Parity by Product Name

eFigure 1. Changes in Orally-Administered Anticancer Medication Use Per 100,000 Enrollees Pre- and Post-Parity by Funding Status and Out-of-Pocket Spending Cap Use

eFigure 2. Changes in Mean Annual Prescription Drug Spending by Health Plans Pre-and Post-Parity by Plan Funding, and Presence Versus Absence of an Out-of-Pocket Spending Cap

eTable 2. Changes in the Distribution of Out-of-Pocket Spending Per Fill Pre-and Post-Parity by Plan Funding, Stratified by Whether Caps Applied Pre- and Post-Deductible or Post-Deductible Alone (Among States with Caps)

This supplemental material has been provided by the authors to give readers additional information about their work.

© 2021 Dusetzina et al. *JAMA Health Forum*.

eMethods. Description of Propensity Score Weighting Approach

In addition to accounting for time-invariant characteristics through our modeling approach, we estimated propensity score weighted (adjusted) models, including age group, sex, and the quarter of the year when the prescription was filled using a multinomial logistic regression model to generate the propensity score for each group (pre- versus post-parity; fully-insured versus self-funded; caps versus no caps). We created propensity score weights as 1/probability of treatment for each observation's assigned group. We compared characteristics of groups of interest pre- and post- propensity score weighting using chi-squared tests and t-tests, finding that our groups were balanced on these limited characteristics. As recommended in studies focused on application of difference-in-differences models assessing policy change, we excluded year and the specific drug filled from the propensity score model as these variables are closely tied to group membership (particularly, year) and thus not recommended for inclusion in difference-in-differences models.

© 2021 Dusetzina et al. *JAMA Health Forum*.

eTable 1. Anticancer Medication Fills Observed in the 24 Months Pre- and Post-Parity by Product Name

Drug Name	Number of Fills
Gleevec	28742
Revlimid	27403
Capecitabine	17781
Xeloda	15640
Sprycel	14573
Tasigna	9622
Afinitor	9015
Ibrance	8941
Tarceva	7513
Imbruvica	5790
Zytiga	5573
Votrient	4600
Sutent	4592
Jakafi	3744
Nexavar	3516
Pomalyst	3466
Xtandi	3067
Tykerb	2685
imatinib	2306
Xalkori	2109
Fareston	2013
Tafinlar	1911
Stivarga	1868
Thalomid	1861
Inlyta	1803
Mekinist	1756
Gilotrif	1446
Ofev	1270
Lonsurf	1231
Erivedge	1192
Bosulif	1081
Zelboraf	1051
Lynparza	993
Iclusig	812
Targretin	672
Zykadia	643
Ninlaro	638
Lenvima	636

Drugs with fewer than 500 fills during the study period were included in the analysis but not separately listed in the Table (Afinitor, Alecensa, Alunbrig, Bexarotene, Cabometyx, Calquence, Caprelsa, Cometriq, Cotellic, Farydak, Hexalen, Hycamtin, IDHIFA, Impavido, Iressa, Kisquali, Lysodren, Nerlynx, Nilandron, Odomzo, Rubraca, Rydapt, Tagrisso, Venclexta, Verzenio, Zejula, Zolinza, Zydelig).

© 2021 Dusetzina et al. *JAMA Health Forum*.

eFigure 1. Changes in Orally-Administered Anticancer Medication Use Per 100,000 Enrollees Pre- and Post-Parity by Funding Status and Out-of-Pocket Spending Cap Use

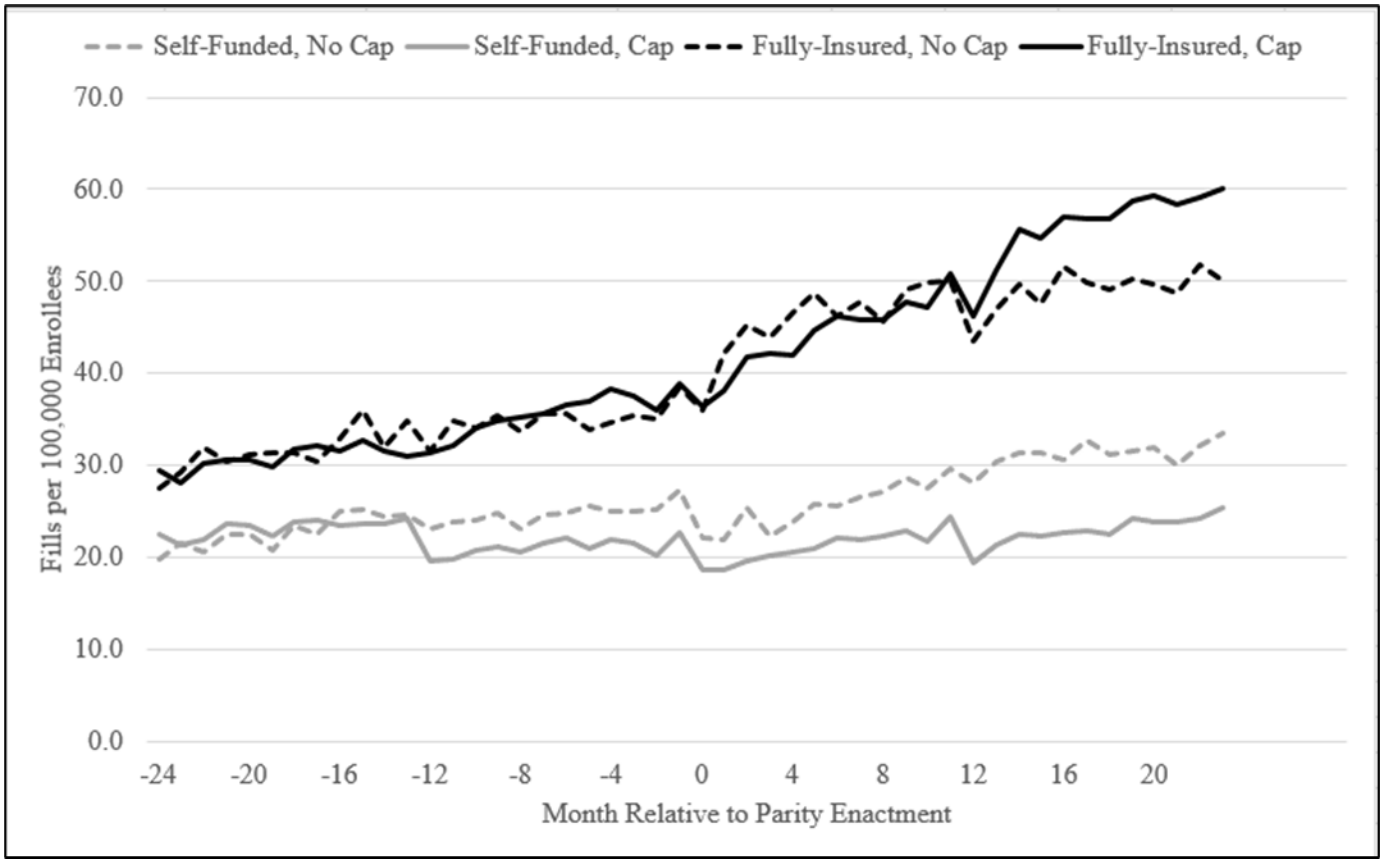

Authors analysis of 2011-2017 HCCI claims data. We did not find evidence for differential baseline trends pre-parity, supporting the parallel trends assumption (Trend by cap and plan type: 0.10, 95%CI: -0.01, 0.21).

© 2021 Dusetzina et al. *JAMA Health Forum*.

eFigure 2. Changes in Mean Annual Prescription Drug Spending by Health Plans Pre-and Post-Parity by Plan Funding, and Presence Versus Absence of an Out-of-Pocket Spending Cap

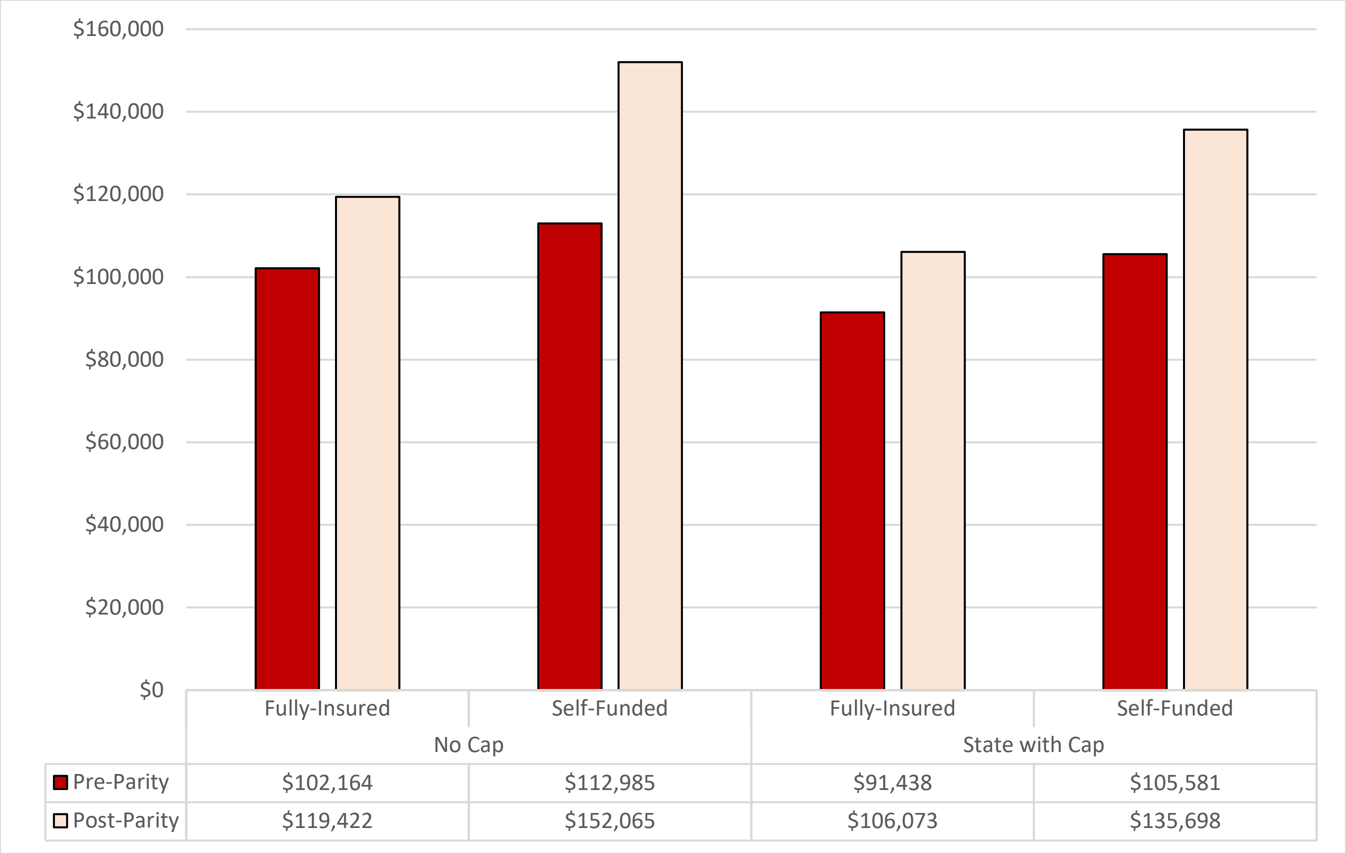

Authors analysis of 2011-2017 HCCI claims data. Means were estimated using PROC GENMOD with an identity link and normal distribution in SAS 9.4. Propensity score weighted DDD models estimated a non-statistically significant additional $8,856 per person-year in annual total drug spending for those in fully-insured plans with caps relative to those in fully-insured plans without caps, controlling for changes among self-funded members over the same period (DDD: $8,856, 95%CI:-$5579, $23291; p=0.23).

© 2021 Dusetzina et al. *JAMA Health Forum*.

eTable 2. Changes in the Distribution of Out-of-Pocket Spending Per Fill Pre-and Post-Parity by Plan Funding, Stratified by Whether Caps Applied Pre- and Post-Deductible or Post-Deductible Alone (Among States with Caps)

	Fully-Insured		Self-Funded		DD Estimate: Fully-Insured versus Self-Funded			
Cap Applied Pre- and Post- Deductible	Pre Parity n= 22,019	Post Parity n= 40,418	Pre Parity n= 12,713	Post Parity n= 12,761	Unadjusted DD	Adjusted DD	95% CI	p-value
Mean	$304	$198	$150	$172	-$126	-$130	(-$179, -$80)	<0.001
25th Percentile	$0	$0	$22	$0	$22	$22	--	--
50th Percentile	$39	$0	$44	$31	-$25	-$25	(-$25, -$25)	<0.001
75th Percentile	$216	$76	$83	$65	-$121	-$122	(-$125, -$119)	<0.001
90th Percentile	$607	$527	$111	$139	-$105	-$112	(-$118, -$105)	<0.001
95th Percentile	$1,868	$714	$218	$377	-$1,315	-$1,313	(-$1,352, -$1,273)	<0.001
Cap Applied Post- Deductible Only	Pre Parity n= 18,562	Post Parity n= 25,579	Pre Parity n= 15,555	Post Parity n= 15,138	Unadjusted DD	Adjusted DD	95% CI	p-value
Mean	$243	$210	$129	$133	-$33	-$37	-$64, -$10	0.01
25th Percentile	$4	$0	$11	$0	$9	$7	--	--
50th Percentile	$39	$0	$33	$20	-$26	-$26	(-$26, -$26)	<0.001
75th Percentile	$97	$77	$61	$56	-$17	-$16	(-$19, -$13)	<0.001
90th Percentile	$518	$530	$133	$132	$16	$12	(-$1, $26)	0.07
95th Percentile	$1,135	$735	$281	$306	-$408	-$425	(-$523, -$327)	<0.001

Source: Authors analysis of Health Care Cost Institute Claims, 2011-2017.
*Means were estimated using PROC GENMOD with an identity link and Gamma distribution. Quantile regression was estimated using PROC QUANTREG in SAS 9.4.
Models were adjusted using inverse probability of treatment propensity score weights.

© 2021 Dusetzina et al. *JAMA Health Forum*.
